# Clinical Outcomes and Medical Burdens of Neonatal Arrhythmias in Children’s Hospitals in China: A Protocol for Multi-Center Retrospective Cohort Study

**DOI:** 10.1007/s00246-024-03421-z

**Published:** 2024-02-19

**Authors:** Jia Na, Songwei Wu, Lu Chen, Yujie Qi, Yue Yuan, Guoshuang Feng, Xinyu Wang, Mingyan Hei

**Affiliations:** 1 Department of Cardiology, Beijing Children’s Hospital Capital Medical University, National Center for Children’s Health, Beijing, 100045 China; 2 Department of Neonatal Center, Beijing Children’s Hospital Capital Medical University, National Center for Children’s Health, Beijing, 100045 China; 3 Big Data and Engineering Research Center, Beijing Children’s Hospital Capital Medical University, National Center for Children’s Health, Beijing, 100045 China

**Keywords:** Neonatal arrhythmias, Heart failure, Risk factors, Prognosis, Medical burden

## Abstract

Neonatal arrhythmias are significant contributors to infant mortality. Timely diagnosis and treatment are essential for neonates with non-benign arrhythmias to avoid severe complications, and ongoing treatment and follow-up are sometimes needed. The main objective of this study will be to understand the incidence and demographic characteristics of arrhythmias in hospitalized neonates in China and the related factors of outcomes. A secondary objective will be to establish the first follow-up system for neonatal arrhythmias in China. The medical burdens of neonatal arrhythmias in China will also be investigated. The data from the Futang Research Center of Pediatric Development (FRCPD) database between January 2016 and December 2021 were obtained. Newborns admitted to member hospitals with a discharge diagnosis of “neonatal arrhythmia” (ICD-10 code P29.151) or “arrhythmia” (ICD-10 code I49.904) were included. The medical record information was collected and classified into two groups: heart failure and non-heart failure. The differences between the two groups and independent risk factors for neonatal arrhythmias complicated with heart failure were analyzed. In addition, a follow-up study of patients discharged from Beijing Children’s Hospital was conducted to evaluate their outcomes at the age of 3 years old. Factors influencing hospitalization costs were analyzed using rank-sum tests and multiple linear regression. It is anticipated that the study findings will provide new and comprehensive data on the health needs of neonatal arrhythmias in China. The study will establish the first follow-up system for neonatal arrhythmias in China. This study will help reduce the burden of patients and their families as well as the society.

## Introduction

Cardiac arrhythmia is a significant cardiovascular disorder in the neonatal period and can result in infant mortality if not diagnosed or treated promptly. The incidence of arrhythmia is about 0.1% to 4.8% during the neonatal period [1]. In neonatal intensive care units (NICUs), the incidence of cardiac arrhythmia could reach 10% [1]. Approximately 1% to 3% of fetal cardiac arrhythmias were detected during pregnancy [2]. Neonatal arrhythmia is a heterogeneous disease, and its severity of onset is influenced by various factors. In many hospitals in China, clinicians often struggle to accurately identify and promptly manage neonatal cardiac arrhythmia, leading to referrals to higher-level hospitals. Therefore, the significance of studying neonatal arrhythmia and corresponding healthcare burden should not be overlooked.

Traditionally, neonatal arrhythmia has been classified into benign and non-benign according to the severity of clinical manifestations [3, 4]. Benign arrhythmias generally exhibit no significant symptoms and do not require special treatment as long as hemodynamics remain stable. Non-benign arrhythmias, however, typically present with symptoms and necessitate timely treatment to avoid severe complications and poor prognosis. Cardiac specialists commonly classify arrhythmias as tachyarrhythmias and bradyarrhythmias [5]. Premature atrial contractions (PACs) are the most common benign arrhythmia in newborns, which usually do not require special treatment [1, 6, 7]. On the other hand, supraventricular tachycardia (SVT), including paroxysmal supraventricular tachycardia (PSVT) and atrial tachycardia (AT), as well as ventricular tachycardia (VT), are the more common non-benign tachyarrhythmias in the neonatal period. Complete atrioventricular block (AVB) is a severe non-benign bradyarrhythmia. Drug therapy is the preferred treatment for the aforementioned neonatal tachyarrhythmias. The first choice for treating neonatal arrhythmia is medication. But prolonged use of drugs may have certain side effects for some children in need of long-term oral medication. The effectiveness of drug treatment varies and is affected by patient compliance. In addition, the choice of antiarrhythmic drugs may be limited if there is heart failure. In these cases, catheter radiofrequency ablation (RFA) is considered. In 2016, the Pediatric and Congenital Electrophysiology Society in collaboration with the Heart Rhythm Society published an expert consensus on pediatric RFA [8]. Recommendations for the indications of RFA for pediatric tachyarrhythmias were provided, including recurrent or sustained SVT with associated impaired cardiac function and ineffective medical treatment; symptomatic SVT patients weighing ≥ 15 kg; patients weighing ≥ 15 kg with ventricular pre-excitation causing pre-excitation cardiomyopathy, with ineffective medical treatment or intolerance; and recurrent monomorphic VT with associated impaired cardiac function, all of which are considered Class I recommendations for catheter ablation. According to the Chinese growth curve standards for preschool children [9], the 50th percentile weight for a 3-year-old child is 15 kg. Therefore, apart from weight restrictions, the age of over 3 years old is considered the clinical empiricism safe age for RFA in children. Factors such as smaller blood vessels and cardiac chamber volume make RFA more challenging and complex in younger and lower-weight infants. However, recent studies have shown that the incidence of catheter ablation complications in lower-weight patients is not higher [10–12]. Therefore, the decision of whether to choose RFA for these patients remains controversy.

Sustained episodes of tachyarrhythmias, such as SVT and VT, can lead to hemodynamic instability and congestive heart failure, in which long-term use of antiarrhythmic drugs may be necessary. Following the neonatal period, these patients require ongoing treatment and follow-up with pediatric cardiology specialists. Regular monitoring through electrocardiograms, Holter monitoring, and echocardiograms are essential to timely evaluate efficacy and detect adverse reactions. Some patients may ultimately require RFA [13]. Additionally, parents or caregivers of affected children need to invest more time and effort into their daily care, which require education during hospital discharge or outpatient visits [14]. Congenital complete AVB in patients with structurally normal hearts is often associated with maternal autoantibodies that damage the atrioventricular node through placental transfer [15]. These patients also require long-term follow-up in pediatric cardiology clinics, with many of them in need of lifelong pacemaker therapy. Thus, the establishment of a follow-up system for neonatal arrhythmia is crucial, particularly for patients with non-benign arrhythmias. The primary objective is to guide medication usage and provide timely catheter ablation for eligible patients. Considering the significant financial and time burdens of long-term treatment for patients with non-benign arrhythmia, research on healthcare burden is also necessary.

The Futang Research Center of Pediatric Development (FRCPD) is the first non-profit organization in China dedicated to research in pediatric development. The FUTang Updating medical REcords (FUTURE) database facilitates the sharing of medical data resources [16]. As of May 2023, a total of 24 member hospitals (all tertiary children’s medical institutions at the provincial and municipal levels, distributed across 21 provinces/autonomous regions/municipalities in seven regions of China) within the FRCPD have agreed to upload the home-page information of inpatient medical records to the FUTURE database. A research team, comprising experienced physicians from the Neonatology Center and Pediatric Cardiology Department of Beijing Children’s Hospital (BCH), along with professional methodologists from the FUTURE Big Data Center, aims to utilize FUTURE database to understand the demographic characteristics, types, and outcomes of neonatal arrhythmia in China. This will serve as the foundation for the establishment of the registration and management platform for the first neonatal arrhythmia database in China, as well as a patient follow-up system. The Pediatric Cardiology Department of BCH, designated as a “Training Base for Cardiac Arrhythmia Intervention (Catheter Ablation)” by the National Health Commission has achieved favorable outcomes in young, low-weight infants and complicated cases with arrhythmia. FUTURE database was estimated to include over 2000 newborns with a discharge diagnosis of “neonatal arrhythmia” or “arrhythmia” over a 6-year period. During the same period, approximately 250 cases of neonatal arrhythmia was treated in BCH, with 150 cases per year in children under 3 years old with arrhythmia at the Pediatric Cardiology Department. Based on the follow-up information, the research team has established a preliminary follow-up system for patients with neonatal arrhythmia in a single center to understand the outcomes of arrhythmia at 3 years of age. This will lay the foundation for further individualized treatment and follow-up management, with plans for future multi-center implementation. The establishment of such a system can promote the precision and standardization of medical treatment for pediatric arrhythmias and cardiac interventions in China owing to lack of such system. Moreover, this study aimed to investigate the healthcare burden of neonatal arrhythmia in the current socio-economic development status of China, providing a basis for hospitals to control costs and alleviate financial burden on patients and their families.

## Methods

### Study Design

This is a multi-center retrospective cohort study.

### Study Population

The study population comprises newborns admitted to the neonatology department, including NICU, of 24 FRCPD member hospitals between January 1, 2016, and December 31, 2021.

### Inclusion Criteria

(1) Age at admission ≤ 28 days. (2) Hospitalized and treated in the neonatology department/neonatal intensive care unit of FRCPD member hospitals. (3) Discharge diagnosis including neonatal arrhythmia (ICD-10 code P29.151) or arrhythmia (ICD-10 code I49.904).

### Exclusion Criteria

(1) Cases with incomplete initial information that cannot be verified. (2) Cases transferred during hospitalization.

### Data Collection and Quality Control

Data will be collected from the home-page information database and will include the following: (1) Demographic characteristics: full-term/premature, birth weight, gender, ethnicity, region, history of perinatal asphyxia, age at admission. (2) General clinical information: admission time, mode of admission, comorbidities (heart failure, respiratory failure, congenital heart disease including congenital heart malformation, atrial septal defect, ventricular septal defect, and patent ductus arteriosus), evidence of infection, discharge outcomes (cured, improved, not cured, death). (3) Types of arrhythmias: Benign arrhythmias (e.g., sinus bradycardia, premature atrial contractions, premature junctional contractions, premature ventricular contractions) and non-benign arrhythmias (e.g., paroxysmal supraventricular tachycardia, Wolff-Parkinson-White syndrome, atrial tachycardia, atrial flutter, atrial fibrillation, junctional tachycardia, ventricular tachycardia, ventricular flutter, ventricular fibrillation, sinus node dysfunction, second-degree or higher atrioventricular block, long QT syndrome). (4) Disease burden information: length of hospital stay, hospitalization costs, proportion of medical insurance payment, and cost structure.

This study would be carried out as a multi-center retrospective study based on the data from medical records. Considering the difference in follow-up process among these medical centers, follow-up details could not be described. The follow-up duration would be 3 years. Follow-up of discharged patients from BCH up to the age of 3 years old will involve collecting data through telephone interviews or outpatient visits. This will include clinical symptoms, echocardiograms, electrocardiograms or Holter monitoring, and details of medication or cardiac interventional treatment which included both of pacemaker placement or ablation.

The selection and determination of data indicators will be carried out by pediatric cardiologists and neonatologists. Dedicated statisticians with professional background of clinical medicine at the FUTURE Big Data Center will perform data verification and cleaning. Data will be collected and organized in an anonymized manner. Data security measures will adhere to the regulations for electronic medical record security and confidentiality in each hospital.

To reduce bias, homogeneity analysis will be conducted for each hospital. Hospital variables include bed size, annual number of discharged cases, gestational age and birth weight of discharged cases, proportion of disease spectrum, ratio of doctors to nurses, ratio of beds to doctors, average length of stay, and annual mortality in the neonatal ward.

### Definitions

Neonatal cardiac arrhythmia encompasses diseases identified by the discharge diagnosis codes P29.151 or I49.904 according to the ICD-10 classification [17].

Concomitant infectious diseases are diagnosed based on the electronic case home-page information in the FUTURE database. These diagnoses include neonatal sepsis/septicemia, neonatal meningitis/encephalitis, neonatal viral myocarditis, neonatal pneumonia, digestive system infection, urinary tract infection, skin infection, and infections specific to the perinatal period. Causality is not differentiated.

Comorbidity with heart failure refers to cases whom heart failure is not initially diagnosed at admission but is included in the discharge diagnosis. The data of heart failure (P29.001) or respiratory failure (P28.501) are collected according to the definitions in ICD-10 codes.

Adverse prognosis in discharge outcomes encompasses treatment abandonment (indicating that the child did not meet the discharge criteria and treatment was terminated with the legal guardian’s signature) and death (the mortality rate is calculated by dividing the number of deaths during hospitalization by the number of hospitalizations in the same period).

The “cured” included both cured with medications and without medications. For patients with bradyarrhythmias, “cured” is defined as heart rate of not lower than the normal minimum value for the same age and without atrioventricular conduction block.

“Improved” means that the patient’s vital signs were stable. Arrhythmias are effectively controlled (whether or not antiarrhythmic drugs are used) for patients with “tachycardia.” For children with bradyarrhythmias without indications for pacemaker implantation, vital signs stabilize and there are no episodes of As Syndrome after internal medicine treatment. For children with concurrent heart failure, there is an improvement in heart function, with a modified Ross classification of heart function at level I or II.

Those with discharge diagnosis of “After pacemaker implantation” is identified as who receive pacemakers.

### Statistical Methods

Data analysis will be conducted using *JMP Pro* (version 16) statistical software. Descriptive statistics will be employed to characterize the cohort. Number and proportion will be used for count data, while mean, standard deviation, median, interquartile range (25th and 75th percentiles), maximum value, and minimum value will be used for continuous data. Normally distributed continuous data will be presented as mean ± standard deviation (x ± sd), and independent samples t tests will be used for group comparisons. Non-normally distributed continuous data will be presented as M (P_25_, P_75_), and the *Mann–Whitney U* test will be employed for group comparisons. Count data will be presented as *n* (%), and group comparisons will be performed using the chi-square test. In cases where the test requirements are not met, *Fisher’s exact* test will be applied. Univariate analysis of hospitalization costs will be conducted using the *Wilcoxon rank-sum* test. The *Wilcoxon rank-sum* test will be used for comparisons between two groups, and the *Kruskal–Wallis H* test will be used for comparisons among multiple groups. Multiple linear regression analysis will be performed to identify factors influencing hospitalization costs. A significance level of *P* < 0.05 will be considered statistically significant.

### Ethical Review

This study was approved by the Clinical Medical Ethics Committee of BCH (approval number: 2020-k-10), and informed consent was waived.

## Results

The collected data, statistical measurements, and corresponding *p* values will be presented in tabular format.

### Primary Outcome Measures

The total number of neonatal cases hospitalized during the study period, the number of included cases with neonatal arrhythmias, and the number of deaths during hospitalization will be recorded. The proportion of neonatal arrhythmia cases and the mortality rate during the study period will be calculated. The demographic information, general clinical information, types of arrhythmias, and discharge outcomes of the included patients will be analyzed. The demographic characteristics of the patients, comorbidities, and the classification and frequency of different arrhythmia types will be examined. The demographic information, general clinical information, types of arrhythmias, and discharge outcomes of patients with concomitant infectious diseases will be compared between neonates with arrhythmias with heart failure and those without heart failure. The results will be presented using bar graphs, pie charts, and other appropriate visualizations.

The disease burden information of the included patients will be summarized, including the median length of hospital stay, median hospitalization costs, proportion of medical insurance coverage, and the composition of hospitalization costs (such as the proportion of bed fees, medication costs, laboratory and examination fees, and surgical fees). For data from multicenters, only the data of “Ancillary examination fees” would be available. The “ancillary testing” would be available in data collected from Beijing Children’s Hospital.

### Secondary Outcome Measures

Variables showing statistically significant and clinically meaningful results from the univariate analysis of demographic and general clinical information will undergo further multiple logistic regression analysis to identify independent risk factors for neonatal arrhythmias complicated with heart failure. Sex, age at admission, premature birth status, and the presence of infection will be included as covariates.

After adjusting for potential confounders, multiple linear regression will be used to investigate the factors influencing inpatient costs for neonatal arrhythmias, as well as the magnitude of their effects.

### Patient Follow-Up

Case report forms were completed. The follow-up process is illustrated in the flowchart below (Table 1).

**Table 1:** Patient follow-up flowchart

Data indicators	Screening	Follow-up
Follow-up time window	Admission	At 3 years of age
Inclusion criteria	✔	
Diagnosis of arrhythmia	✔	
Clinical symptoms	✔	✔
Electrocardiogram/Holter (if applicable)	✔	✔
Cardiac echocardiography (if applicable)	✔	✔
Medication information	✔	✔
Cardiac intervention treatment		✔
Wearable arrhythmia technology	✔	

### Subgroup Analysis of Beijing Children’s Hospital

The data form multi-center would not be available because they were collected from medical records. But the medication data from one of those center (Beijing Children’s Hospital) would be available; thus, a subgroup analysis would be conducted.

## Discussion

Current understanding of the clinical characteristics, long-term prognosis, and resulting medical burden of neonatal arrhythmias is not adequate due to the unique nature of the newborn population and the complexity of cardiac arrhythmia. It is anticipated that the study findings will provide new and comprehensive data on the health needs of neonatal arrhythmias in China.

### Study Feasibility

FUTURE database provides a data-sharing platform for home-page information of inpatient medical records, covering a total of 24 member hospitals (distributed across 21 provinces/autonomous regions/municipalities in seven regions of China). The multi-center retrospective cohort study design ensures the security and confidentiality of the participants. The Research Office of BCH provides study governance, ensuring that safe research methodologies are implemented. The research team members include experienced physicians from the Neonatology Center and Pediatric Cardiology Department of BCH, along with professional methodologists from the FUTURE Big Data Center.

### Study Limitations

The laboratory values, imaging pictures, medications, and certain clinical variables of the cases are not available. The data form multi-center will not be available because they were collected from medical records. But the medication data from one of those center, BCH would be available; thus, a subgroup analysis would be conducted. The investigators are highly dependent on the accuracy and completeness of initial data entry of each hospital case.

## Conclusions

The study will increase our understanding of the demographic and clinical characteristics and the associated medical costs of neonatal arrhythmias in China. The study will establish the first follow-up system for neonatal arrhythmias in China. This will lay the foundation for further individualized antiarrhythmic treatment and follow-up management of newborns and infants, with plans for future multi-center implementation. The results may also provide resource for policy and practice applicable to control costs and alleviate the financial burden on patients and society.

### Safety Considerations

As this study is retrospective in nature, it does not involve any additional laboratory tests or pose additional risks to the patients. Only the patients’ clinical data will be collected, and their identifiable information will be removed prior to statistical analysis to ensure privacy. The study was approved by the Medical Ethics Committee of BCH, Capital Medical University, as mentioned above, and waived from informed consents.

### Confidentiality

The data uploading and formatting procedures adhere to the standards set by the Hospital Quality Monitoring System (HQMS) for collecting first-page information from inpatient medical records in the *Performance Appraisal and Medical Quality Management of National Tertiary Public Hospitals* (2019). The quality control plan for the FUTURE database includes de-identification, coding instructions, and data cleaning. During the de-identification process, any potentially identifying information about the patients or their parents was masked or deleted before the data were uploaded. Diagnosis, histology, and operation coding were conducted using standardized international classifications such as the International Classification of Diseases (10th Revision), International Classification of Diseases for Oncology (3rd Edition), and International Classification of Diseases 9th Revision Clinical Modification Operations and Procedures 3rd edition (ICD-9-CM-3). Following code standardization, data cleaning procedures were implemented to ensure the uniqueness, integrity, and validity of each record [16, 18].

### Data Access

Access to the raw dataset will be restricted to the statistician and the principal investigator.

### Dissemination Policy

The findings of this study will be submitted for publication in peer-reviewed scientific journals and will also be presented at local, national, and international conferences for dissemination.

## Data Availability

All data generated or analyzed during this study are included in this published article.

## Declarations

### Research Involving Human and Animals Rights

There was no involvement of patients and/or the public in the design, conduct, reporting, or dissemination plans of this research study.
